# *STOPGAP*: an open-source package for template matching, subtomogram alignment and classification

**DOI:** 10.1107/S205979832400295X

**Published:** 2024-04-12

**Authors:** William Wan, Sagar Khavnekar, Jonathan Wagner

**Affiliations:** a Department of Biochemistry and Center for Structural Biology, Vanderbilt University, Nashville, TN 37240, USA; b Max Planck Institute of Biochemistry, Martinsried, Germany; National Centre for Biological Sciences-TIFR, India

**Keywords:** *STOPGAP*, cryo-ET, open source software, subtomogram alignment

## Abstract

Cryo-electron tomography (cryo-ET) enables molecular-resolution 3D imaging of complex biological specimens such as viral particles, cellular sections and, in some cases, whole cells. This enables the structural characterization of molecules in their near-native environments, without the need for purification or separation, thereby preserving biological information such as conformational states and spatial relationships between different molecular species. Subtomogram averaging is an image-processing workflow that allows users to leverage cryo-ET data to identify and localize target molecules, determine high-resolution structures of repeating molecular species and classify different conformational states. Here, *STOPGAP*, an open-source package for subtomogram averaging that is designed to provide users with fine control over each of these steps, is described. In providing detailed descriptions of the image-processing algorithms that *STOPGAP* uses, this manuscript is also intended to serve as a technical resource to users as well as for further community-driven software development.

## Abstract 2

*STOPGAP*, an open-source package for subtomogram averaging that is designed to provide users with fine control over each of the steps in the image-processing workflow, is described.

## 1. Introduction

Cryo-electron microscopy (cryo-EM) combined with single-particle analysis (SPA) has in recent years become a key method for determining the structures of biological macromolecules (Kühlbrandt, 2014 ▸). Ideal SPA specimens consist of a monolayer of purified molecules suspended in vitreous ice, with each molecule producing a randomly oriented projection in the resultant electron micrographs. SPA determines structures by iterating between aligning each projection to a 3D reference structure and reconstructing an improved reference structure using the new alignment parameters. One limitation of SPA is that the specimen typically needs to consist of a monolayer of particles, otherwise the molecular projections begin to overlap one another in the micrographs, thereby hindering alignment and structure determination.

There are a number of situations where overlapping structures cannot be avoided, including pleomorphic assemblies, membrane-associated complexes and molecules in near-native cellular environments (Beck & Baumeister, 2016 ▸). In these situations, key structural and biological information is inextricably tied to the complexity of the molecular environments, making the separation, isolation or purification of molecular components undesirable. Cryo-electron tomography (cryo-ET) offers one approach to solving the problem of imaging overlapping molecules. In cryo-ET, rather than collecting a single image for each field of view, each field of view is imaged multiple times while tilting the specimen stage. This set of 2D projections, called a tilt series, is then used to directly reconstruct a 3D representation of the field of view: a tomogram.

While tomograms overcome the overlap problem, they still suffer from a number of fundamental limitations. Cryo-ET specimens typically consist of either biological assemblies frozen into holey support grids, similar to single-particle cryo-EM specimens, vitrified cells or cellular sections, such as focus ion beam (FIB)-milled lamella. In each case, specimens have a roughly slab-like profile; when tilted, these slab-like specimens effectively become thicker with respect to the electron beam. This increasing thickness, coupled with physical restrictions of the microscope hardware, limits the angular range over which tomographic data can be collected. A common tilt range is from −60° to 60°, resulting in a specimen that is effectively twice as thick at the extreme tilt angles. Incomplete angular sampling, due to limitations in both the overall tilt range as well as the angular increments between tilt images, leads to unsampled or missing regions of Fourier space; this is referred to as the ‘missing wedge’ (Schmid & Booth, 2008 ▸). While the missing wedge refers to unsampled regions of Fourier space, it also produces corresponding distortions in real space. Additionally, any other factors that affect electron micrographs also affect tomograms, such as the contrast transfer function (CTF; Wade, 1992 ▸; Fernández *et al.*, 2006 ▸; Xiong *et al.*, 2009 ▸; Turoňová *et al.*, 2017 ▸) and electron exposure damage (Grant & Grigorieff, 2015 ▸), which fundamentally limit the amount of high-resolution information that can be obtained. As such, high-resolution structure determination from tomograms still requires the averaging of repeating structures across a data set in order to fill the missing wedge, flatten CTF modulations and increase the signal-to-noise ratio.

Determining structures from cryo-ET data requires a wide range of processing steps (Wan & Briggs, 2016 ▸; Schur, 2019 ▸; Castaño-Díez & Zanetti, 2019 ▸; Leigh *et al.*, 2019 ▸; Zhang, 2019 ▸; Pyle & Zanetti, 2021 ▸). This includes steps to process raw 2D data into 3D reconstructions, such as tilt-series alignment, defocus determination, CTF correction and tomographic reconstruction. The series of processing steps that starts after tomographic reconstruction and ends with the generation of higher resolution averaged EM density maps is analogous to SPA and is typically referred to as subtomogram averaging (STA). STA includes a number of tasks such as 3D particle picking, often by template matching, the generation of higher resolution EM density maps by iterative subtomogram alignment and averaging, and subtomogram classification to separate heterogeneous structures.

Here, we describe *STOPGAP*, an open-source package for STA. *STOPGAP* uses a real-space correlation-based approach similar to a number of other STA packages (Förster *et al.*, 2005 ▸; Hrabe *et al.*, 2012 ▸; Nicastro *et al.*, 2006 ▸; Castaño-Díez *et al.*, 2012 ▸; Himes & Zhang, 2018 ▸; Ni *et al.*, 2022 ▸), but contains several new algorithms that we describe here. These real-space correlation-based approaches are distinct from the Bayesian approach of *RELION* (Scheres, 2012 ▸; Bharat *et al.*, 2015 ▸; Zivanov *et al.*, 2022 ▸), which compares references and subtomograms in Fourier space using the *RELION* regularized likelihood function. This function is used to calculate the probabilities of each orientation for each subtomogram; these probability values are then used to generate probability-weighted averages. At the core of many of the *STOPGAP* algorithms is the treatment of the missing wedge, which improves the performance of template matching and sub­tomogram alignment, as well as the quality of averaged EM density maps. We also describe algorithms for subtomogram classification by multi-reference alignment (MRA) that use stochastic approaches which allow an assessment of the reproducibility of classification results.

## 2. Methods

### 2.1. Software overview

*STOPGAP* is an open-source package written in MATLAB consisting of a main *STOPGAP* executable, which is run as a compiled MATLAB executable, and the ‘toolbox’, a set of MATLAB functions and scripts. The *STOPGAP* executable is supplied pre-compiled, but can also be compiled by end users. Example bash scripts for running *STOPGAP* in parallel using the message-passing interface (MPI) or SLURM Workload Manager are provided, although these will likely need to be edited to match specific cluster configurations.

In addition to the main executable, a ‘parser’ executable is also supplied, which checks for conflicting parameters and generates properly formatted parameter files for the various *STOPGAP* tasks. *STOPGAP* tasks include subtomogram extraction, template matching and subtomogram alignment. MRA-based classification is not a separate task but instead is a user-implemented workflow using the subtomogram-alignment task.

### 2.2. Missing-wedge model

The missing wedge is modeled as a series of Fourier slices, each of which can have additional amplitude modulations corresponding to the local CTF of each subtomogram and the exposure filter applied during tomogram reconstruction. For template matching, the CTF filter is calculated as a single global filter that is consistent throughout the tomogram. Local per-particle CTF filters are used for subtomogram alignment, averaging and classification. Local CTF filters are calculated by first assuming that the defocus estimated from a tilt series represents the defocus at the center of mass of the specimen (Turoňová *et al.*, 2017 ▸). The center of mass of the tomogram is estimated using the particle positions in the ‘motive list’, which stores the alignment parameters of each subtomogram. For each Fourier slice, the local defocus of the subtomogram is calculated using the estimated defocus, the center of mass and the distance from the tilt axis in each tilt projection.

### 2.3. Constrained cross correlation

Constrained cross correlations (CCCs) are calculated using a 3D version of the fast local correlation function (FLCF; Roseman, 2003 ▸; Castaño-Díez *et al.*, 2012 ▸). The FLCF is a real-space correlation function that normalizes the reference map (*i.e.* reference or template) and the search map (*i.e.* tomogram or subtomogram) with respect to the mask applied to the reference. Prior to calculation of the FLCF, reference maps are filtered with the amplitude-modulated missing-wedge filter, while search maps are filtered with binary slice wedge masks to remove any reconstruction-related or cropping-related Fourier space artifacts.

### 2.4. Template matching

During template matching, each tomogram is split into sub­volumes, termed ‘tiles’, for parallel processing. For each tile, the template is rotated through a set of orientations defined in the ‘angle list’ and a CCC is calculated from the rotated template and tile. The highest scoring voxels for each orientation are accumulated into a ‘score map’, while associated orientations are stored in the ‘orientation map’. Motive lists are then generated by first thresholding score maps to a user-defined level, followed by a peak-finding algorithm that searches for the highest scoring voxels with a user-supplied inter-peak distance related to the particle dimensions. Particle positions are taken from the peak positions in the score map, while particle orientations are taken from the same positions in the orientation map.

*STOPGAP* template matching also includes an additional noise-correlation approach, similar to that used in calculating Fourier shell correlations (Chen *et al.*, 2013 ▸). In this approach, a noise volume is first generated by randomizing the phases of the template. This noise template is used for matching alongside the actual template, resulting in a noise score map that is used to reweight the main score map. Reweighting has the effect of shifting the distribution of the CCC values downwards. Negative correlations in the reweighted score map are then set to zero, which effectively flattens the noise, allowing better visual analysis for score thresholding.

When using template matching, there are several different parameters that can be optimized by the user. The source of the template heavily influences the performance of the CCC function. Templates that are generated in *STOPGAP* typically work best, as the filtering approaches are internally consistent, but cryo-EM and cryo-ET maps from other packages also work well. Simulated maps using the *UCSF Chimera* *molmap* tool (Pettersen *et al.*, 2004 ▸) typically perform poorly as they are poor representations of EM densities; the *simulate* tool in *cisTEM* (Grant *et al.*, 2018 ▸) performs much better. Iterative approaches where an initial round of template matching using strict thresholding is followed by STA and another round of matching are typically appropriate. We find that template masks contoured to the template typically perform better due to improved normalization. Angular search step and low-pass filtering are interrelated as higher resolution information can only be adequately searched for using finer angles. For a more detailed description of the optimization of template-matching parameters, we refer users to the publication by Cruz-León and coworkers, who have undertaken an extensive analysis using *STOPGAP* template matching (Cruz-León *et al.*, 2023 ▸).

### 2.5. Subtomogram extraction

Subtomogram extraction is performed by cropping sub­volumes from each tomogram according to the positions stored in the motive list. The positions in the motive list refer to the center of each subtomogram. The default file format for subtomograms is the .mrc format, while the .em format is also supported; subtomograms saved as .mrc files can be stored as 8-, 16- or 32-bit data.

### 2.6. Subtomogram alignment and averaging

Subtomogram alignment revolves around the parameters stored in the motive list, primarily the CCC score, shift and rotation values from prior iterations. Input for an alignment round includes bandpass filter settings, angular increments, which define the granularity of the search space, and angular iterations, which define the size of the search space. The angular increments and iterations are used to calculate a list of search orientations with respect to the prior determined orientation. At each orientation the CCC is calculated; the peak value is taken as the CCC score, while the vector from the center of the CCC map to the peak is taken as the shift vector. A cross-correlation mask can be applied to limit the shifts allowed. As alignment progresses, the angular increments are decreased to finer angles to align higher resolution features. After alignment is completed, a new motive list with updated CCC scores, shifts and rotations is produced. This new motive list is then used to average new references.

Subtomogram alignment and averaging is always performed as ‘halfsets’ where the motive list is split into two and aligned and averaged independently. For so-called ‘gold-standard’ alignment (Scheres & Chen, 2012 ▸), where halfsets are aligned and averaged completely independently, the halfset of each subtomogram must be defined in the motive list. If halfsets are not defined, *STOPGAP* will randomly split the data set in half during each iteration. At the end of the averaging process, three output volumes are written out: the two halfmaps and a figure-of-merit weighted (Rosenthal & Henderson, 2003 ▸) sum of the halfmaps. The figure-of-merit weighted map is provided for easy evaluation of the STA iteration as the two halfmaps can appear quite noisy prior to low-pass filtering at the estimated resolution; in the *STOPGAP* workflow, low-pass filtering and sharpening is carried out independently of subtomogram alignment and averaging.

Initial particle picking using approaches such as template matching provides relatively precise starting orientations. As such, the subtomogram-alignment process primarily deals with local refinement of the orientations. The main parameters here are the binning of the tomographic data, the angular increment and range, and the low-pass filter settings. While the number of iterations is also a parameter, typically alignment converges within two iterations with a given parameter set. High-resolution cryo-ET data are typically collected with a pixel size between 1 and 2 Å and are processed using binning factors of 8×, 4×, 2× and 1×; unbinning to the next step is appropriate when the estimated resolution is beyond the Nyquist resolution. The initial angular search range should be slightly wider than the angular step used in template matching. From there, the angular error is of the order of the angular increment used. We typically find that a 2–3° angular increment with an angular iteration of 3 (*i.e.* a total search range of ±6–9°) is sufficient until the final phase of alignment. As a typical example, for template matching using 15° steps, a 3° angular increment and six angular iterations per iteration will sufficiently align the subtomograms for a localized search. From there, 2–3° angular increments and three angular iterations can be used until the solution converges at unbinned data; finer increments and smaller iterations can then be used for final alignments. The low-pass filter should be adjusted whenever the alignment parameters change. We typically adjust these conservatively, setting the low-pass filter radius where the FSC measures at least 0.9 until the final stages of alignment. The low-pass filter radius is then incremented until FSC equals 0.5; at this point we consider the alignment to be complete.

### 2.7. Classification by MRA

In standard single-reference alignment, *STOPGAP* is essentially performing a 3D search of orientation space, with the 3D translation implicitly determined as the peak position in the CCC map. MRA is formulated as a 4D search, with the fourth dimension denoting the references or classes. As such, the set of search orientations not only contains the three Euler angles, but also each reference. Standard subtomogram alignment takes the form of an expectation-maximization or hill-climbing algorithm, where the complete set of search orientations is scored and the maximum-scoring orientation is accepted. *STOPGAP* alignment includes a stochastic hill-climbing (SHC) approach inspired by similar SPA approaches (Reboul *et al.*, 2016 ▸), where the previous best orientation is scored first while the rest of the orientations are scored in random order. An orientation that scores better than the previous orientation is immediately accepted and *STOPGAP* moves on to the next subtomogram. SHC ensures that better scoring orientations are found at each iteration, but avoids becoming trapped in local optima by not searching for the ‘best’ orientation. An additional simulated-annealing (SA) method is also implemented on top of the SHC approach, which allows the acceptance of worse-scoring orientations. In SA, after scoring the previous best orientation, a worse-scoring orientation can be accepted with a given input probability; the annealing occurs by decreasing this probability to zero over subsequent iterations.

*De novo* reference generation refers to the process of generating multiple references from the data set without *a priori* knowledge of the class of each subtomogram. This is typically performed on a subset of the data by first deciding on the number of classes to be produced and then randomly splitting the data set into even bins for each class, followed by MRA. To minimize the potential for initialization bias, *STOPGAP* can initialize *de novo* references by asking the user for the number of desired classes and the number of subtomograms per class. It then randomly selects subtomograms to populate each class in a non-exclusive manner. This is different from evenly splitting the data set as a subtomogram may appear in multiple classes or none during initialization, reducing the potential and impact of the attractor problem (Sorzano *et al.*, 2010 ▸).

Following *de novo* reference generation, MRA is then performed on the initial references to refine them into divergent structures. MRA is first performed using SA, which has the effect of increasing the particle movement between classes, followed by SHC until convergence. The stochastic methods in *STOPGAP* have the additional benefit that MRA can be performed with the same parameter set to produce different results, which we use as a way of judging the reproducibility of our classification. Repeated MRA runs that produce classes with consistent sizes suggest stable separation of conformations as well as providing an estimate of the error of class occupancies. Likewise, subtomograms that repeatedly find the same class are likely to be true members of that class, while subtomograms that converge on to different classes with each MRA run suggests that they are bad particles that can be removed from the data set.

For computationally efficient classification by MRA, we generally recommend aligning the subtomograms as a single class up to 2× binning, which typically provides resolutions better than 20 Å. It is best to align by masking the structurally invariant part of the average; in the case of ribosomes we first align on the large subunit. From there, we can perform alignment with no angular search and use a mask that focuses on the variable regions of the structure; for example, the ribosomal tRNA channel. We typically recommend starting with more classes than are thought to be present. This improves the likelihood of finding classes with smaller occupancies, but may result in some classes representing the same structure; these can be merged later on. *De novo* reference generation and MRA can be performed and final classes can be merged as necessary after visual analysis. At least three repeats of *de novo* reference generation and MRA should be performed, and final classes can be assigned for subtomograms that are consistently classified in the majority repeats; sub­tomograms that are not consistently classified can be removed from the data set. After the final classes have been assigned, sub­tomogram alignment can be performed for each class independently using the *ali_multiclass* mode of *STOPGAP* to obtain final high-resolution maps. See below for the procedure used for the ribosome data set described in this manuscript.

### 2.8. 80S ribosome preprocessing and tomogram reconstruction

Tomographic data preprocessing and reconstruction was performed using *TOMOgram MANager* (*TOMOMAN*; Khavnekar, Erdmann *et al.*, 2023 ▸). EER images were motion-corrected using the *RELION* implementation of *MotionCor* (Zivanov *et al.*, 2018 ▸) and exposure filtered (Grant & Grigorieff, 2015 ▸) in *TOMOMAN*. Defocus was estimated using the *tiltCTF* module in *TOMOMAN*, which calculates tilt-adjusted periodogram averages that are then processed by *CTFFIND*4 (Rohou & Grigorieff, 2015 ▸). Tilt series were aligned using fiducial-less alignment in *ARETOMO* (Zheng *et al.*, 2022 ▸). 3D CTF-corrected tomograms were generated with *NovaCTF* and binned by Fourier cropping (Turoňová *et al.*, 2017 ▸).

### 2.9. 80S ribosome template matching

Template matching was performed on 8× binned, 3D CTF-corrected tomograms. The *Saccharomyces cerevisiae* 80S ribosome template was generated from atomic coordinates (PDB entry 6gqv) using the *simulate* tool in the *cisTEM* package (Grant *et al.*, 2018 ▸) at the unbinned pixel size. This was then binned to 8× and low-pass filtered to 30 Å with a final box size of 32 pixels. Template matching was performed with 15° angular spacing, resulting in 4512 orientations. Template matching was performed with a low-pass filter cutoff of 13 Fourier pixels using CTF and exposure filtering and noise correlation. Score maps were manually thresholded, resulting in 269 987 particles from 200 tomograms.

### 2.10. 80S ribosome subtomogram alignment and classification

The 8× binned motive list generated from template matching was rescaled to 4× and subtomograms were extracted with a 64-pixel box size. Subtomogram alignment was performed using a mask contoured to the full ribosome density. Two iterations were performed with a 5° angular increment and three angular iterations, followed by two iterations with a 3° angular increment and three angular iterations; the low-pass filters for these iterations had cutoffs of 18, 22, 26 and 28 Fourier pixels, respectively. After these iterations, the CCC scores showed a bimodal distribution and the data set was thresholded with a cutoff of 0.165, resulting in 100 750 remaining particles. These subtomograms were further aligned for one iteration with a 2° angular increment, three angular iterations and a low-pass filter cutoff of 28 Fourier pixels.

The final 4× binned motive list was rescaled to 2× binning and extracted with a 128-pixel box size. Two iterations of alignment were performed with a 2° angular increment and three angular iterations, followed by one iteration using a 1° angular increment and three angular iterations; the low-pass filter cutoffs were 32, 45 and 48 Fourier pixels, respectively. The first two iterations used a mask focused on the whole ribosomal density, while the last two iterations used a mask focusing on the large subunit density.

For classification, initial *de novo* references were generated from the final 2× binned motive list with ten classes, each containing 2% of the total data set per class, picked using the randomization approach described above. The alignment mask used for classification was contoured to isolate the ribosomal tRNA channel. MRA was performed with ten iterations of simulated annealing with a temperature range from 10 to 0°C, followed by 30 iterations of alignment with SHC; no angular search was used. All iterations were performed with a low-pass filter cutoff of 45 Fourier pixels. Three replicates of MRA were performed using the same parameters.

Classes were visually inspected and those representing the same ribosomal states were merged, resulting in five distinct states. Particles were then assigned by class consensus, where particles that found the same class two out of three times were kept, while those that did not were removed. This resulted in a final data set of 84 443 subtomograms.

After classification, orientations for each class were independently refined with two iterations with a 5° angular increment, three angular iterations and a low-pass filter cutoff of 45 Fourier pixels, followed by two iterations with a 3° angular increment, three angular iterations and a low-pass filter cutoff of 45 Fourier pixels.

### 2.11. HIV s-CANC preprocessing and tomogram reconstruction

Processing of the five-tomogram subset (tilt series 1, 3, 43, 45 and 54) of EMPIAR-10164 was performed using the *TOMOMAN* package. Frame alignment was performed using *MotionCor*2 (Zheng *et al.*, 2017 ▸), followed by exposure filtering in *TOMOMAN* and tilt-series alignment in *IMOD* using fiducial-based alignment (Kremer *et al.*, 1996 ▸). After tilt-series alignment, defocus estimation was performed using the *tiltCTF* module in *TOMOMAN*. 3D CTF-corrected tomograms were reconstructed using *novaCTF* and Fourier cropped to 2×, 4× and 8× binnings.

### 2.12. HIV s-CANC initial reference generation

For initial *de novo* reference generation, the centers of nine s-CANC assemblies were manually picked in *UCSF Chimera* (Pettersen *et al.*, 2004 ▸). Initial subtomogram positions were generated along the spherical surfaces with a three-pixel spacing, resulting in 25 876 subtomograms. Initial cone angles were calculated based on the position on the spherical surface, while in-plane angles were randomized.

Subtomograms were extracted with a 32-pixel box size. To generate an initial structure, four iterations of subtomogram alignment were performed with *C*1 symmetry, a spherical mask, an out-of-plane search with a 3° angular increment and three angular iterations, an in-plane search with a 10° angular increment and six angular iterations, and a low-pass filter radius of six Fourier pixels. To shift the *C*6 symmetry axis onto the volume *Z* axis, the shift vector was first estimated by visual analysis and then applied to the motive list. After this, all alignment was performed using *C*6 symmetry. After shifting, a new reference was averaged and further aligned for three iterations using an out-of-plane search with a 3° angular increment and three angular iterations, an in-plane search with a 3° angular increment and four angular iterations, and a low-pass filter radius of six Fourier pixels. The motive list was then cleaned to remove overlapping particles using a center-to-center distance of five voxels and particles with CCC values under 0.5; this resulted in 4040 remaining particles. In-plane angles were randomized around the *C*6 symmetry (*i.e.* in random increments of 60°) and aligned for one more iteration using an out-of-plane search with a 3° angular increment and three angular iterations, an in-plane search with a 3° angular increment and four angular iterations, and a low-pass filter radius of six Fourier pixels.

This reference was then used to realign against the initial motive list again, this time using one iteration with *C*6 symmetry, an out-of-plane search with a 3° angular increment and three angular iterations, an in-plane search with a 3° angular increment and 20 angular iterations, and a low-pass filter radius of six Fourier pixels. The motive list was again cleaned using a center-to-center cutoff of five voxels and a CCC cutoff of 0.5; this resulted in 4451 remaining particles. These particles were further refined with two iterations of alignment using a 2° angular increment, three angular iterations and a low-pass filter radius of six Fourier pixels.

### 2.13. HIV s-CANC template matching

Using the 8× binned initial reference, template matching was performed on 8× binned 3D CTF-corrected tomograms using a 10° angular step and a low-pass filter cutoff of six Fourier pixels. Threshold values for score maps were determined by visually assessing each map; this resulted in 21 637 subtomograms.

After template matching, the subtomograms were aligned against the 8× binned starting reference with two iterations using a 3° angular increment, four angular iterations and a low-pass filter radius of six Fourier pixels.

### 2.14. HIV s-CANC high-resolution STA

The 8× binned motive list was rescaled to 4× binning and subtomograms were extracted with a box size of 64 pixels. Subtomograms were aligned for four iterations using a cylindrical mask, *C*6 symmetry, a 2° angular increment and three angular iterations; the low-pass filter cutoffs were six, 12, 18 and 24 Fourier pixels, respectively.

The 4× binned motive list was rescaled to 2× binning and subtomograms were extracted with a box size of 128 pixels. Subtomograms were aligned for five iterations using a cylindrical mask, *C*6 symmetry, a 2° angular increment and three angular iterations; the low-pass filter cutoffs were 24, 30, 36, 42 and 48 Fourier pixels, respectively.

The 2× binned motive list was rescaled to 1× binning and subtomograms were extracted with a box size of 256 pixels. Subtomograms were first aligned for three iterations using *C*6 symmetry, a cylindrical mask, a 2° angular increment and three angular iterations; the low-pass filter cutoff was 48 Fourier pixels, but the last iteration included a high-pass filter of five Fourier pixels. Another iteration was performed using these settings but with a contoured mask that focused on the central hexamer and its immediate neighbors. Another five iterations were performed using the contoured mask, a 1° angular increment and two angular iterations; the low-pass filter cutoffs were 48, 56, 62, 68 and 68 Fourier pixels, respectively, with a high-pass filter of five Fourier pixels. One additional iteration was performed using a 0.5° angular increment and two angular iterations followed by another iteration with a 0.5° angular increment and one angular iteration; a low-pass cutoff of 68 Fourier pixels and a high-pass cutoff of seven Fourier pixels were used for both iterations.

The centers of mass for each tomogram were calculated by taking the mean of the *Z* positions of the subtomograms in each tomogram; these were used to refine the tomogram centers during 3D CTF-corrected tomogram reconstruction in *novaCTF*. Subtomograms were extracted with a box size of 256 pixels and aligned using a contoured mask for one iteration using a 1° angular increment, two angular iterations, a low-pass cutoff of 68 Fourier pixels and a high-pass cutoff of seven Fourier pixels. Next, four iterations were performed with a 0.5° angular increment and two angular iterations; low-pass cutoffs of 68, 68, 74 and 80 Fourier pixels, respectively, and a high-pass cutoff of seven Fourier pixels were used. Next, two iterations of alignment were performed with a 0.5° angular increment, two angular iterations, a low-pass cutoff of 80 Fourier pixels, a high-pass cutoff of seven Fourier pixels and CCC thresholdings of 0.06 and 0.07, respectively. A final round of alignment was performed with a 0.25° angular increment, two angular iterations, a low-pass cutoff of 84 Fourier pixels, a high-pass cutoff of seven Fourier pixels and a CCC thresholding of 0.07.

## 3. Results

### 3.1. Missing-wedge model

Distortions caused by the missing wedge effectively result in structural features that are not present in the specimen or the isotropically resolved reference maps used in STA. As such, real-space comparisons between anisotropic tomographic data and isotropic references can lead to poor results such as imprecise subtomogram alignment. To overcome this, real-space correlation STA packages apply a missing-wedge filter to references prior to computing the cross correlation with tomographic data; this is referred to as the constrained cross correlation (CCC; Förster *et al.*, 2005 ▸; Frangakis *et al.*, 2002 ▸; Schmid & Booth, 2008 ▸; Bartesaghi *et al.*, 2008 ▸). The missing-wedge filter is a Fourier-space filter that is meant to mimic the effects of tilt-series collection and tomographic reconstruction, *i.e.* the anisotropic sampling, in order to reproduce the real-space tomographic distortions in the isotropically resolved reference. In real-space STA packages that use CCCs, the missing-wedge filter is typically generated as a binary filter that includes all information between the maximum and minimum tilt angles (Förster *et al.*, 2005 ▸; Hrabe *et al.*, 2012 ▸; Nicastro *et al.*, 2006 ▸; Castaño-Díez *et al.*, 2012 ▸). While this accounts for the missing region caused by the limited tilt range, it does not accurately describe the distortions in a tomographic reconstruction resulting from discrete angular sampling, CTF and electron-exposure damage.

In *STOPGAP*, the missing-wedge filter is modeled to reflect the sampling geometry and amplitude modulations present in the tomographic data. This includes using Fourier slices rather than a solid wedge, CTF modulations and exposure filtering. *STOPGAP* requires CTF correction prior to or during reconstruction using methods such as tilted CTF correction (Xiong *et al.*, 2009 ▸) or 3D CTF correction (Turoňová *et al.*, 2017 ▸; Kunz & Frangakis, 2017 ▸). *STOPGAP* also accounts for aliasing in the amplitude spectrum, which, when combined with CTF correction prior to or during tomogram reconstruction, allows tight cropping of subtomograms to minimize computational costs while avoiding potential signal loss due to signal delocalization outside the subtomogram edges.

The *STOPGAP* missing-wedge model is similar to that implemented in *RELION*3 (Bharat *et al.*, 2015 ▸), although it differs in its calculation and usage. Broadly speaking, both are designed to represent microscope aberrations, beam-induced damage and the tomographic reconstruction, but *STOPGAP* uses its missing-wedge representation as an imaging filter so that the reference contains the same aberrations as the tomographic data prior to computing the real-space cross correlation. As such, *STOPGAP* requires that tilt-series preprocessing and tomographic reconstruction include exposure filtering and 3D CTF correction, as it uses the same parameters to generate the missing-wedge filter. In *RELION*3, the missing-wedge model is an extension of its Bayesian framework, making the CTF and exposure (the dose-dependent *B* factor in *RELION*) parameters optimized during subtomogram alignment. Since *RELION*3 works in Fourier space, the missing-wedge representation effectively acts as an amplitude-modulation function in computing the *l* ^2^-norm. The CTF model is also used for CTF correction, so unlike in *STOPGAP* the input tomograms do not require prior 3D CTF correction. *RELION*4 uses a different pseudo-subtomogram method that approximates direct working on projection data, so the missing wedge is not explicitly accounted for (Zivanov *et al.*, 2022 ▸).

**Figure 1:**
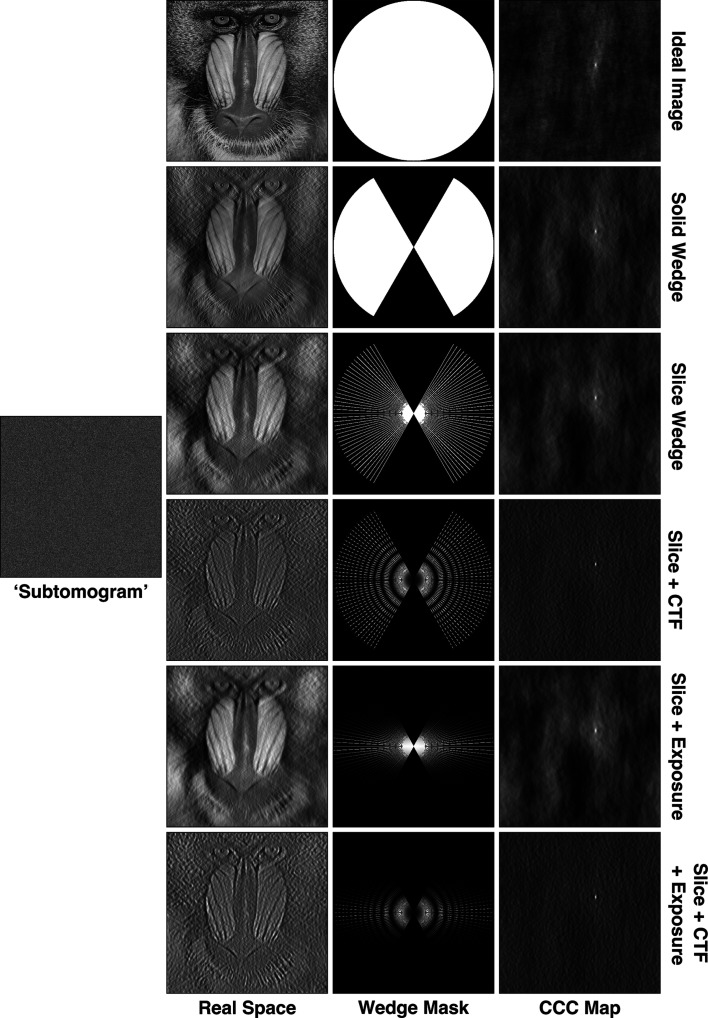
Simulated images with various missing-wedge filters and corresponding CCC maps. The ideal mandrill test image has no filters; the ‘subtomogram’ image is the ideal image shifted towards the upper right corner, with a slice wedge, CTF and exposure filter applied, as well as Gaussian noise at a signal-to-noise ratio of 0.05. The remaining real-space images have the noted missing-wedge filters applied, while the corresponding CCC maps are calculated between the real-space images and the simulated subtomogram.

To illustrate the impact of different missing-wedge components on the CCC, simulated 2D examples are shown in Fig. 1 ▸. These examples represent tomographic planes orthogonal to the tilt axis, *i.e.* the standard *XZ* plane in tomograms. Applying no missing-wedge mask produces a peak in the cross-correlation map, but with significant background. Applying a continuous wedge or a per-tilt-slice wedge produces a slightly sharper peak, but still shows significant background correlation. While the per-tilt-slice wedge has a very similar performance to the continuous wedge mask, the slices also allow the application of tilt-image-specific amplitude-modulating factors such as CTF and exposure filtering.

Amplitude modulations in Fourier space cause signal delocalization in real space, which often leads to high levels of background in CCC space. While CTF correction ensures that amplitude modulations are positive, the sinusoidal modulation is always present. By properly accounting for these amplitude modulations in the wedge mask, *STOPGAP* effectively matches the delocalization between the reference and the tomographic data, resulting in sharp CCC peaks with minimal background noise. To calculate CCCs, *STOPGAP* first filters templates or references by applying a slice-wedge mask with CTF and exposure filter modulations and correlates it to the tomogram or subtomogram using a 3D version of the fast local correlation function (FLCF; Roseman, 2003 ▸; Castaño-Díez *et al.*, 2012 ▸). The benefits of the *STOPGAP* missing-wedge model for different STA tasks are shown below.

### 3.2. Template matching

**Figure 2:**
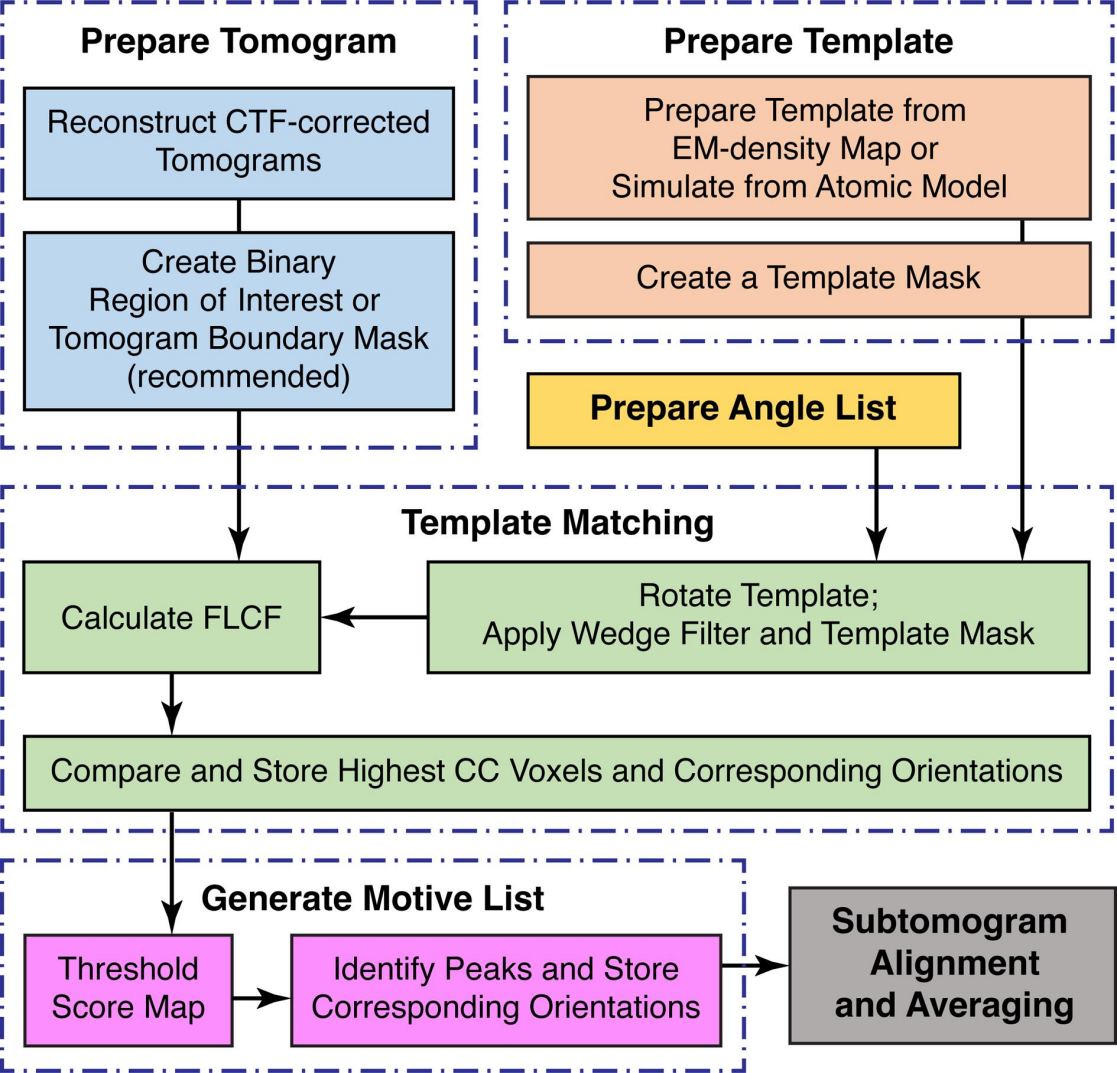
Workflow diagram for template matching. The top row outlines the preparation of the various inputs. The middle row outlines the iterative template-matching process. The bottom row outlines the steps for identifying true peaks in the score maps and how they feed into the next steps of the subtomogram-averaging workflow.

Template matching is a reference-based approach that uses a predetermined reference map, *i.e.* a ‘template’, to identify target particles in tomographic data (Fig. 2 ▸; Frangakis *et al.*, 2002 ▸). Templates can either be EM density maps determined using SPA or STA, or simulated density maps generated from atomic models. During template matching, the template is iteratively rotated through a set of orientations that typically cover all of orientation space with a given angular step size (Figs. 2 ▸ and 3 ▸). At each iteration, CCCs are calculated between the template and the tomogram of interest; high-valued voxels in the CCC map indicate a potential match for the template in that orientation and position. In the first orientation, the CCC map is stored as a cumulative score map, along with a corresponding orientation map, which stores the template orientation at each tomographic position. While iterating through each orientation, new CCC maps are compared with the score map; voxels with the highest values are stored in the score maps and the corresponding orientations are updated in the orientation map. After all orientations have been sampled, the final score map can then be thresholded and the peak positions and their corresponding orientations are taken as potential particles.

**Figure 3:**
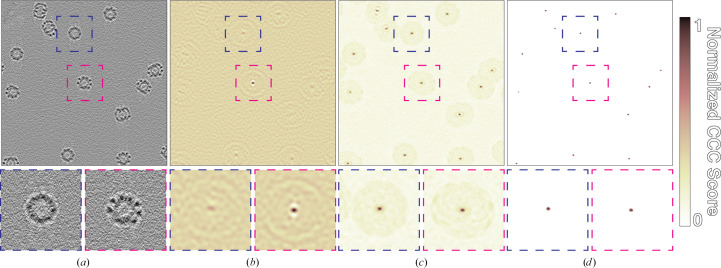
Simulated template matching of thermosomes. (*a*) Slice through a simulated tomogram containing randomly placed and oriented thermosomes (PDB entry 3j1b). (*b*) Score map from matching with a single orientation. The peak boxed in magenta shows an ideal match with a strong CCC peak; the peak boxed in blue does not match well and has a correspondingly small peak. (*c*) Final score map after full orientational search. (*d*) Score map from (*c*) thresholded to remove background so that only true peaks remain. The color map shows the normalized CCC score, where 1 denotes the maximum value in the map and 0 denotes the lowest value in the map.

**Figure 4:**
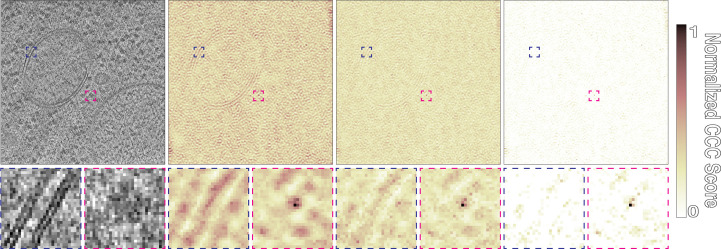
Comparison of template matching in *STOPGAP* with different filters. (*a*) Slice through a tomogram taken from *S. cerevisiae* lamella (EMPIAR-11658). Ribosome template matching with a 15° angular step with (*b*) no CTF or exposure filter, (*c*) CTF and exposure filtering and (*d*) CTF and exposure filtering and noise correlation. Blue boxes indicate CCC scores around a membrane bilayer, while magenta boxes indicate scores around a true positive. The color map shows normalized CCC score, where 1 denotes the value at the CCC peak and 0 the lowest value in the map; the score maps in (*b*), (*c*) and (*d*) have not been thresholded.

Ideally, true positives in the score maps should appear as sharp peaks (Fig. 3 ▸ *d*). However, template-matching results without amplitude-modulation filters tend to show a high level of background correlation, with CCC value distributions that resemble the input maps (Figs. 4 ▸ *a* and 4 ▸ *b*). This includes high CCC values for dense objects such as ice contamination or distinct features such as membrane bilayers. By accounting for amplitude modulations, the *STOPGAP* missing-wedge filter produces CCC peak profiles that are nearly ideal, minimizing noise and false positives (Figs. 3 ▸ *c* and 4 ▸ *c*). While sharper peaks have improved signal strength, background noise often takes on a speckled appearance that can make sharp positive peaks difficult to distinguish; this can be a problem when determining the appropriate CCC value to threshold by. To aid with visual analysis, we developed a noise-correlation approach in which a phase-randomized version of the template is also used for matching. The resultant noise-correlation map represents nonspecific correlation related to weak structural similarities in the tomogram or template mask-related correlations. The noise-correlation map is subtracted from the score map to provide a noise-flattened score map with more pronounced peaks (Figs. 4 ▸ *c* and 4 ▸ *d*). For more specifics on the parameters that affect the quality of template matching, a rigorous study of this has been performed using *STOPGAP* by Cruz-León *et al.* (2023 ▸).

### 3.3. Subtomogram alignment and averaging

**Figure 5:**
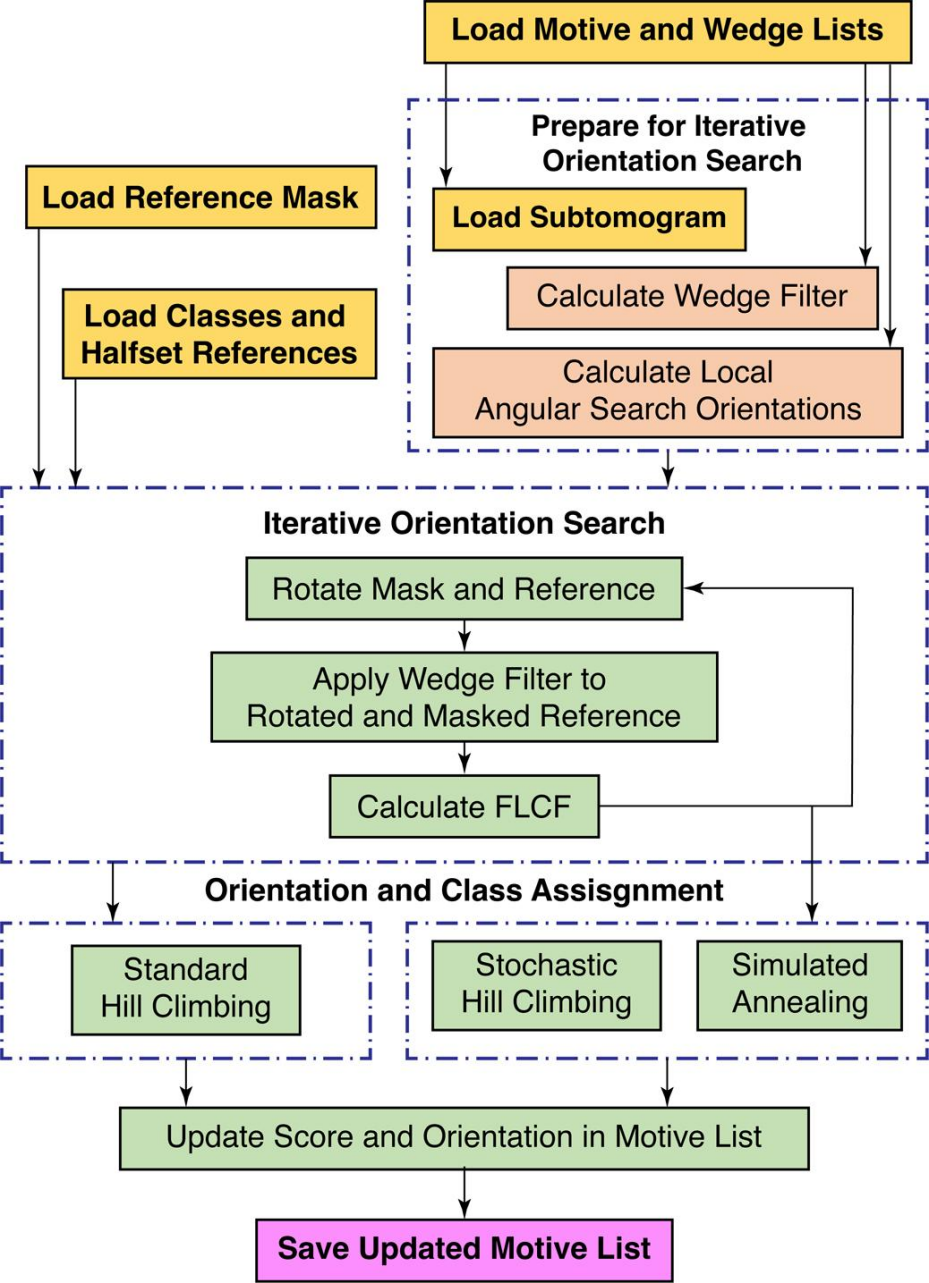
Workflow diagram for subtomogram alignment. The initial loading of the motive and wedge lists, references and reference mask at the top occurs once at the start of the iteration. The remaining steps occur for each entry in the motive list.

**Figure 6:**
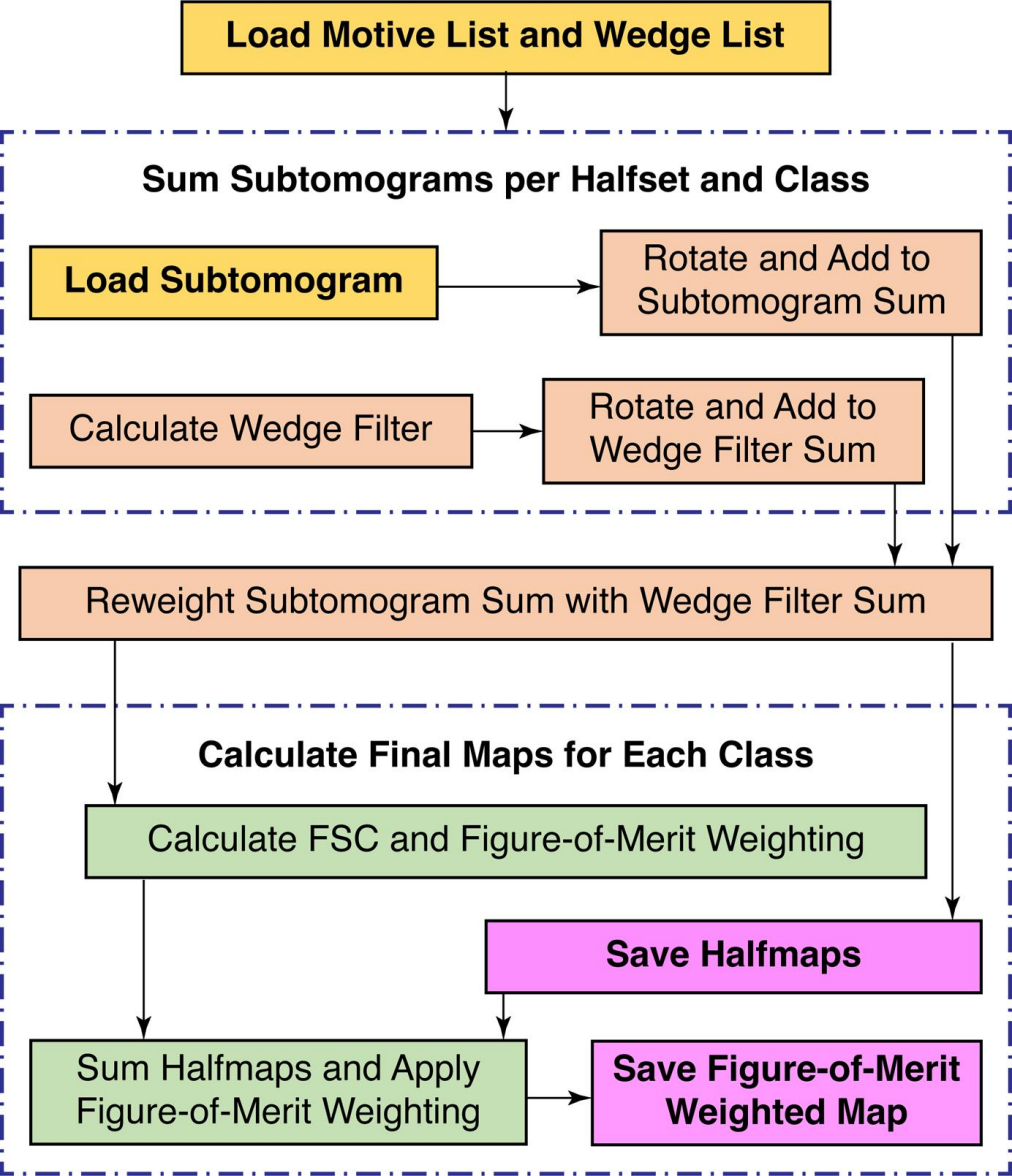
Workflow diagram for subtomogram averaging. This describes the general operation of generating new reference volumes from a given input motive list. This can be performed to generate references prior to alignment or using a new motive list that is output from a round of alignment.

Although we have defined STA broadly as the processing steps that go from tomographic reconstruction up to model building, STA often refers to the determination of higher resolution structures by aligning and averaging subtomograms. Algorithmically, this can be thought of as two steps: the alignment of a reference to a subtomogram to determine the orientation of that particle (Fig. 5 ▸) and the generation of a new reference by averaging subtomograms rotated to their determined orientations (Fig. 6 ▸). Iterating the STA process enables the refinement of subtomogram orientations, as the sub­tomograms are compared with improved references from the prior iteration.

Subtomogram alignment in *STOPGAP* is performed in largely the same way as in most other real-space packages. A reference map is rotated through a set of orientations, which typically represent a local search in orientational space. At each orientation, the CCC is calculated between the rotated reference and a subtomogram (Roseman, 2003 ▸). The maximum value in the CCC map (score) indicates how well the rotated reference matches the subtomogram, while the location of the maximum value in the map provides the Cartesian offset (shift) between the reference and subtomogram. This effectively makes correlation-based STA a 3D rotational search rather than a 6D rotational and translational search, significantly reducing the number of computations. After all orientations have been scored, the orientation with the highest score and its associated shift are taken as the correct subtomogram alignment.

**Figure 7:**
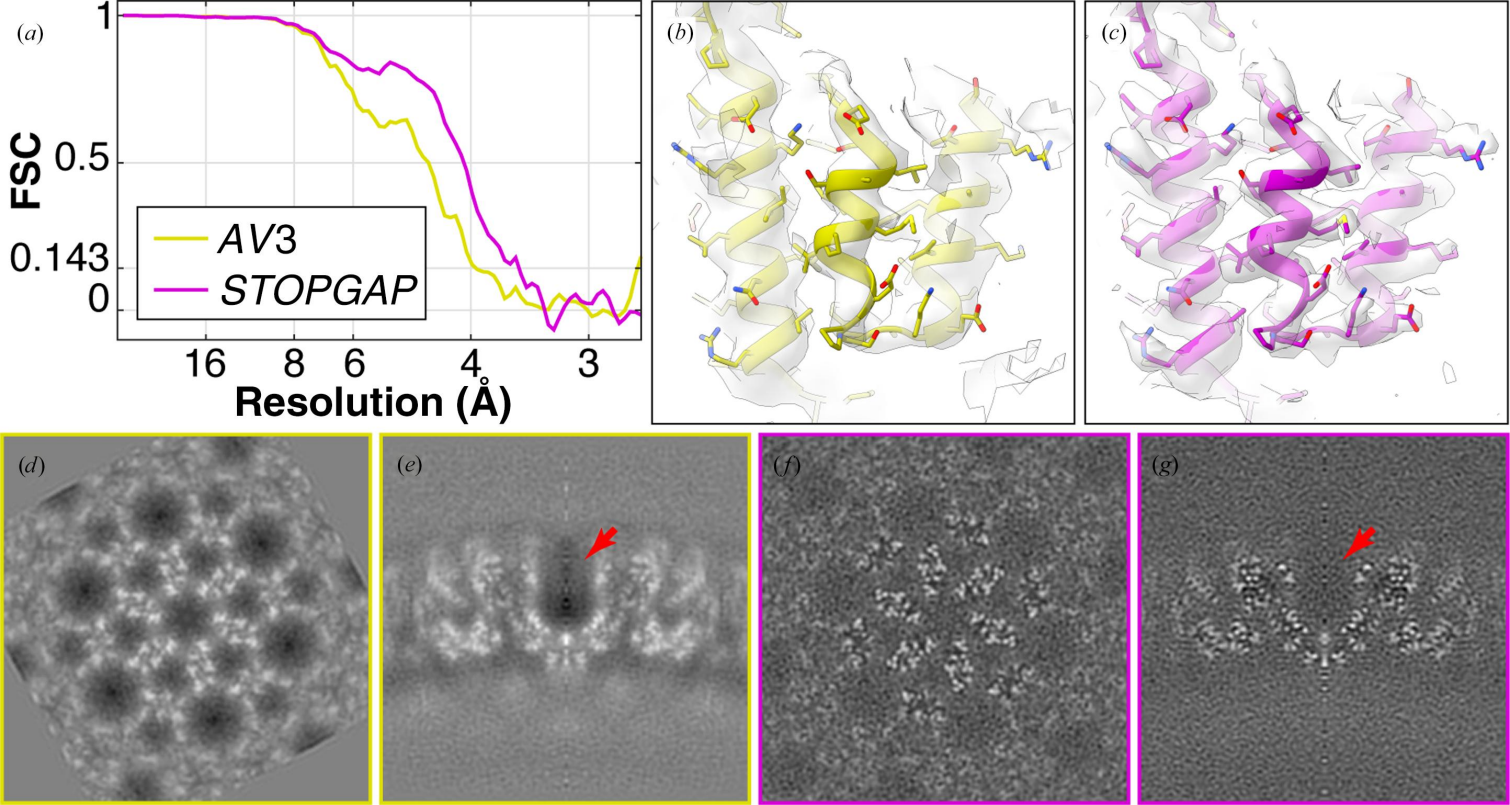
Comparison of HIV s-CANC structures determined using different missing-wedge models. (*a*) Fourier shell correlation (FSC) plots of HIV s-CANC structures determined from the five-tomogram subset of EMPIAR-10164 by *AV*3 (Turoňová *et al.*, 2017 ▸) using a solid wedge mask and *STOPGAP* using CTF and exposure filtering. Both used *novaCTF* (Turoňová *et al.*, 2017 ▸) for 3D CTF-corrected tomogram reconstruction with no tilt-series refinement. (*b*) and (*c*) are example isosurface representations of the *AV*3 and *STOPGAP* EM density maps, respectively. An atomic model (EMD-3782) is rigid-body fitted into both maps. (*d*–*g*) *XY* and *XZ* orthographic slices through the EM density maps determined by *AV*3 and *STOPGAP*, respectively. The red arrow indicates the high negative density region in the *AV*3 map that is not present in the *STOPGAP* map.

We performed subtomogram averaging using the five-tomogram subset of HIV s-CANC particles (EMPIAR-10164) with 3D CTF-corrected tomograms, resulting in a 3.5 Å resolution structure (Fig. 7 ▸). The most directly comparable published structure is the 3.9 Å resolution structure determined using the *AV*3 package (Turoňová *et al.*, 2017 ▸). As with template matching, the *STOPGAP* missing-wedge filter produces sharper CCC peaks that improve the precision of subtomogram alignment, resulting in higher resolution averages. Although higher resolution structures have been determined from this data set using *emClarity*, *M* and *RELION*4 (Himes & Zhang, 2018 ▸; Ni *et al.*, 2022 ▸; Tegunov *et al.*, 2021 ▸; Zivanov *et al.*, 2022 ▸), these structures used tilt-series refinement approaches that are not used here. While tilt-series refinement is not currently implemented in *STOPGAP*, users have already successfully refined *STOPGAP* alignments using *Warp*/*M* or *RELION*4 (Khavnekar *et al.*, 2022 ▸; Rangan *et al.*, 2023 ▸; Schiøtz *et al.*, 2023 ▸).

In addition to improvements to the estimated resolution, the *STOPGAP* missing-wedge filter improves the quality and the interpretability of averaged maps (Figs. 7 ▸ *b*–7 ▸ *g*). This is because after each subtomogram has been rotated, shifted and summed in real space, Fourier-space normalization is required to account for anisotropic sampling (Fig. 6 ▸). This basically accounts for how the missing wedges from each subtomogram combine to fill Fourier space, which is virtually always anisotropic due to incomplete angular sampling in the data set. Fourier normalization is performed by rotating and summing the missing-wedge filter of each subtomogram, producing a map that tallies the per-voxel sampling in Fourier space. By taking amplitude modulations into account, the *STOPGAP* missing-wedge filter produces maps with improved normalization. This is illustrated particularly well in subtomogram averages of HIV s-CANC, where normalization with a binary wedge mask produces strong ‘halos’ of negative densitiy around protein densities (Figs. 7 ▸ *d* and 7 ▸ *e*), a phenomenon that has also been noted by others (Sanchez *et al.*, 2020 ▸). In the *STOPGAP* average, these areas take on a more ideal appearance (Figs. 7 ▸ *f* and 7 ▸ *g*), with gray values around protein densities similar to the surrounding solvent.

### 3.4. Subtomogram classification

Classification in *STOPGAP* is performed using multi-reference alignment (MRA), where each subtomogram is aligned against a set of references, *i.e.* classes. This effectively makes alignment a 4D search problem, with three rotational dimensions and the fourth dimension representing class. References can be from predetermined structures or generated *de novo* from the data set. Given this, we view MRA as two tasks: the initial generation of *de novo* references, typically from a subset of the data set, and the alignment of the full data set against multiple references. In both cases, a key concern is particles becoming trapped in local minima of orientation space. This can be caused by a number of interrelated issues including initialization bias, where a subtomogram ‘finds itself’ in the reference that it contributes to, or premature convergence, where subtomograms stop moving between classes prior to the divergence of structural features in each class.

To overcome these problems, *STOPGAP* has a number of stochastic methods built into its subtomogram-alignment algorithm that can be used during MRA; these are described in detail in Section 2. Briefly, a simulated-annealing (SA) algorithm is used which allows a suboptimal alignment to be accepted with a given probability; this probability is decreased during each iteration of the annealing run. SA encourages more movement of subtomograms between classes during the initial phases of MRA. After SA, a stochastic hill-climbing (SHC) algorithm is used where the previous orientation and class are scored first, and the order of the remaining orientation and class parameters are randomized. The first better scoring orientation and class is immediately accepted, which allows subtomograms to move more during initial iterations and less as the classes take on distinct features. An additional feature of these stochastic methods is that they produce different results when repeating the alignment with the same parameters. We can then use consistency of classification as a rough measure of classification accuracy and precision.

**Figure 8:**
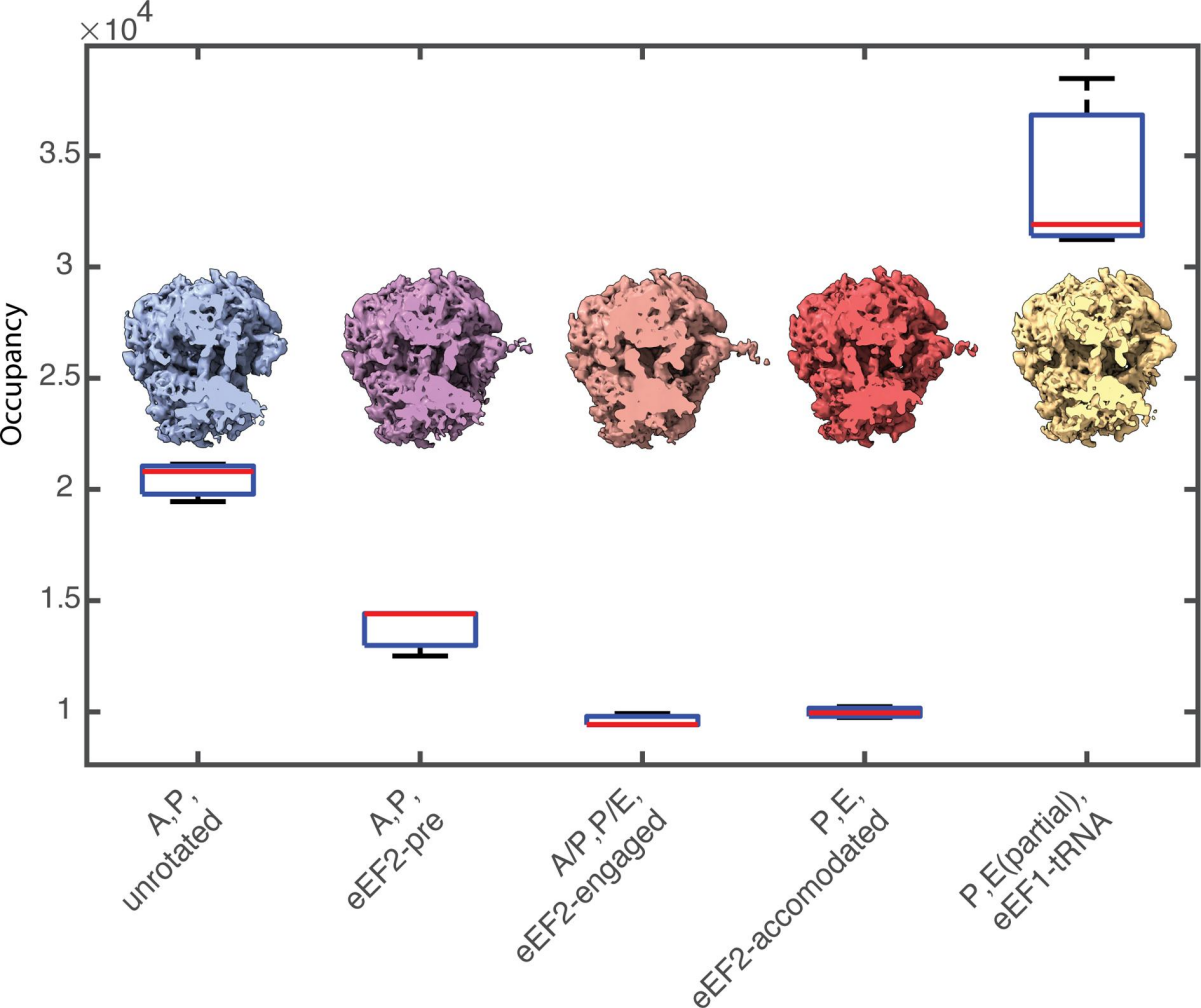
Classification of the *S. cerevisiae* 80S ribosomal translation cycle using MRA. The box plot represents occupancies for each class across three replicates. The red line indicates the sample median, while the bottom and top of each box are the 25th and 75th percentiles of the sample, respectively. Whiskers show the farthest observations beyond the 25th and 75th percentiles. Corresponding density maps for each state after the consensus class assignment are shown with a slice through the tRNA channel. For the ‘P, E(partial), eEF1-tRNA’ state, heterogeneous occupancy of the E-site produces a larger variance across replicates.

To demonstrate the robust multi-reference classification approach in *STOPGAP*, we processed *S. cerevisiae* ribosomes from EMPIAR-11658 (Rangan *et al.*, 2023 ▸). Using the whole 231-tomogram data set, we identified ∼240 000 particles and were able to resolve major structural states of the eukaryotic translation cycle (Fig. 8 ▸).

Initial ribosome positions and orientations were determined using template matching on 8× binned tomograms, resulting in approximately ∼240 000 particles. These were then iteratively aligned at 8× and 4× using a mask shaped to contours of the full ribosome density. Particle scores were distributed bimodally; the ∼100 000 particles in the higher scoring distribution were selected for further processing. These particles were further aligned at 2× binning, first using a full ribosome mask, followed by alignment using a mask focused on the large subunit. The resulting orientations were used as a starting point for a multi-reference alignment. Starting references were generated *de novo* by randomly assigning 20% of the data set to ten classes. To classify the different tRNA states, MRA was performed with a mask focusing on the tRNA channel. The first ten iterations of MRA were performed using SA, followed by MRA with SHC and without SA until convergence, *i.e.* when less than 1% of subtomograms changed classes between iterations. We performed three independent replicates using random *de novo* references and the same MRA parameters. Subtomograms that segregated into the same classes two out of three times were deemed to be consistent and kept, while other particles were deemed to be unstable and discarded. Final classes were assigned by visually curating the final volumes and merging identical states (Supplementary Fig. S1). This resulted in five unique states (Fig. 6 ▸), namely ‘A, P, unrotated’, ‘A, P, eEF2-pre’, ‘A/P, P/E, eEF2-engaged’, ‘E, P, eEF2-accommodated’ and ‘E(partial), P, tRNA-eEF1’ (Milicevic *et al.*, 2024 ▸). Class occupancies between replicates were similar (Fig. 8 ▸), indicating the reproducible separation of stable classes while also providing a metric for quantitating the class assignments. In this instance, we defined class consensus as assignment of a subtomogram to the same class two out of three times. The number of replicates and the stringency for consensus are ultimately defined by the user depending on their specific needs (Erdmann *et al.*, 2021 ▸). Final averages were generated using a consensus subtomogram assignment from each of the three replicate classification runs.

## 4. Discussion

*STOPGAP* is a MATLAB-based open-source package for STA. At its core is a missing-wedge model designed to account for the various types of sampling and modulation effects that occur during tomographic data collection and reconstruction. This missing-wedge model improves the performance of the 3D CCC, which subsequently improves the performance of template matching and subtomogram alignment. This missing-wedge model also improves the Fourier-space normalization function used in STA, providing improved EM density maps. For template matching, we also developed a noise-correlation approach to reduce background correlations in template-matching score maps and enhance the appearance of true peaks. To facilitate MRA, we introduced SA and SHC algorithms into our subtomogram-alignment procedure to help to overcome convergence on local minima as well as to provide metrics for assessing the reproducibility and reliability of subtomogram classifications. Such assessments provide more quantitative values for class occupancies, which is of particular importance for cellular cryo-ET, as quantitative assignment of conformational states is important for characterizing bio­logical states.

Compared with subtomogram alignment, template matching searches a large number of rotations with minimal input/output, making it particularly well suited to GPU acceleration. GPU implementations of template-matching algorithms have recently been added to *PyTOM* (Chaillet *et al.*, 2023 ▸) and *TomoBEAR* (Balyschew *et al.*, 2023 ▸), with *TomoBEAR* using a reimplementation of the *Dynamo* template-matching algorithm (Castaño-Díez *et al.*, 2012 ▸). The performance increase from GPU acceleration enables the use of finer search angles, which enhances the CCC peaks in score maps (Chaillet *et al.*, 2023 ▸; Cruz-León *et al.*, 2023 ▸). To account for CTF modulations, *PyTOM* now applies a CTF filter to the template prior to matching, which produces similar results to the *STOPGAP* missing-wedge model, although the *STOPGAP* per-slice model can account for varying defocus between tilt images and includes exposure filtering. *TomoBEAR* includes additional tools for post-processing the score maps to remove large connected islands of density related to structures such as membranes, although the *STOPGAP* noise-correlation approach often effectively suppresses such false positives. The Turoňová group is currently reimplanting the *STOPGAP* template-matching algorithm with GPU acceleration in their *GAPSTOP* package (https://gitlab.mpcdf.mpg.de/bturo/gapstop_tm), which will greatly improve the computational efficiency over our current implementation.

*STOPGAP* primarily focuses on the aspects of STA that use real-space correlation approaches, with the aim of providing users with fine-grained control of how their data are processed. To this end, an extensive range of parameters are open to the user for fine tuning, although most come with presets that are suitable for a wide range of problems. *STOPGAP* is not intended to be a complete and comprehensive package for all aspects of STA, but instead aims to provide a modular set of tools for carrying out specific image-processing tasks. Given the rich information content of cryo-ET data, we believe that there is no single pipeline that can answer every biological question; the task-oriented approach of *STOPGAP* allows users to tailor pipelines suited to their particular biological questions. Given that *STOPGAP* is unlikely to be the optimal solution to all cryo-ET problems, it is also aimed to be complementary with other cryo-ET packages, enabling users to build comprehensive STA workflows that meet the demands of their specific projects. A number of studies have already been performed using *STOPGAP* as a component of the processing workflow alongside packages such as *Warp*, *M*, *RELION* and *novaSTA* (Hoffmann *et al.*, 2022 ▸; Xing *et al.*, 2023 ▸; Rangan *et al.*, 2023 ▸; Khavnekar *et al.*, 2022 ▸; Khavnekar, Kelley *et al.*, 2023 ▸; Schiøtz *et al.*, 2023 ▸; Turoňová *et al.*, 2020 ▸; Erdmann *et al.*, 2021 ▸; Lacey *et al.*, 2023 ▸). To facilitate cryo-ET data preprocessing and interoperability between STA packages, we are also developing a package called *TOMOMAN*, which handles file-format conversions and directory structures (Khavnekar, Erdmann *et al.*, 2023 ▸). For users who wish to write their own scripts, the *STOPGAP* file formats are described in the documentation and input/output functions are provided in a MATLAB toolbox. Overall, we believe that *STOPGAP* provides a powerful set of image-processing algorithms and tools that are complementary to others in the community and hope that the descriptions of our algorithms will be useful for further community-driven development. *STOPGAP* is available at https://github.com/wan-lab-vanderbilt/STOPGAP.

## Supplementary Material

Supplementary Figure. DOI: 10.1107/S205979832400295X/vo5015sup1.pdf
