# Supplementary material for: STOPGAP: an open-source package for template matching, subtomogram alignment and classification
Source: Acta Crystallogr D Struct Biol. 2024 Apr 12;80(Pt 5):336–49. doi: 10.1107/S205979832400295X (PMC11066880; doi:10.1107/S205979832400295X)
Supplement: Supplementary file 1 [file d-80-00336-sup1.pdf]

STRUCTURAL BIOLOGY

Volume 80 (2024)

Supporting information for article:

STOPGAP: an open-source package for template matching, subtomogram alignment and classification

William Wan, Sagar Khavnekar and Jonathan Wagner

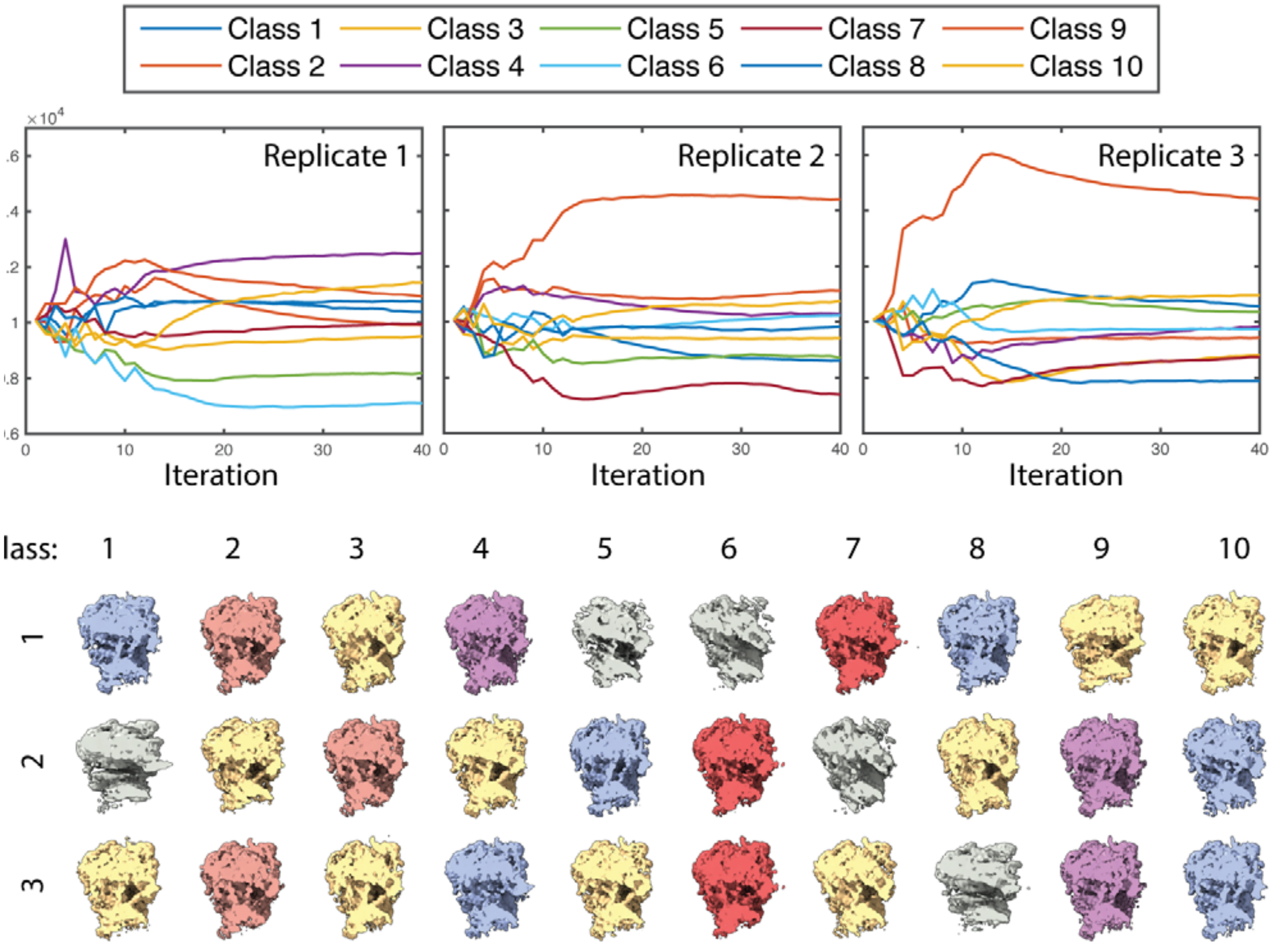

Figure S1 Classification of the *S. cerevisiae* 80S ribosome over three replicates. A) Occupancy of classes over each iteration for each of the three iterations. Class numbers are prior to final curation and are arbitrary between replicates. B) Final density maps after MRA classification with 10 classes across three replicates. Density maps are colored by the visually curated and assigned states depicted in figure 6.
